# High-Accuracy Intermittent Strabismus Screening via Wearable Eye-Tracking and AI-Enhanced Ocular Feature Analysis †

**DOI:** 10.3390/bios15020110

**Published:** 2025-02-14

**Authors:** Zihe Zhao, Hongbei Meng, Shangru Li, Shengbo Wang, Jiaqi Wang, Shuo Gao

**Affiliations:** School of Instrumentation and Optoelectronic Engineering, Beihang University, Beijing 100191, China; by1917059@buaa.edu.cn (Z.Z.); 22371194@buaa.edu.cn (H.M.); lishangru01@gmail.com (S.L.); wangshengb@buaa.edu.cn (S.W.); katrina0625@buaa.edu.cn (J.W.)

**Keywords:** eye tracking, strabismus screening, wearable biosensor, pupil-canthus vectors, machine learning

## Abstract

An effective and highly accurate strabismus screening method is expected to identify potential patients and provide timely treatment to prevent further deterioration, such as amblyopia and even permanent vision loss. To satisfy this need, this work showcases a novel strabismus screening method based on a wearable eye-tracking device combined with an artificial intelligence (AI) algorithm. To identify the minor and occasional inconsistencies in strabismus patients during the binocular coordination process, which are usually seen in early-stage patients and rarely recognized in current studies, the system captures temporally and spatially continuous high-definition infrared images of the eye during wide-angle continuous motion, and is effective in inducing intermittent strabismus. Based on the collected eye motion information, 16 features of the oculomotor process with strong physiological interpretations, which help biomedical staff understand and evaluate results generated later, are calculated through the introduction of pupil-canthus vectors. These features can be normalized, and reflect individual differences. After these features are processed by the random forest (RF) algorithm, this method experimentally yields 97.1% accuracy in strabismus detection in 70 people under diverse indoor testing conditions, validating the high accuracy and robustness of the method, and implying that the method has strong potential to support widespread and highly accurate strabismus screening.

## 1. Introduction

Strabismus is a vision disorder characterized by the inability of the eyes to properly align when focusing on objects [1], gradually leading to amblyopia [2], visual field defects [3], and impaired binocular vision [4]. Its incidence is as high as 4%, especially in children, and it has a genetic predisposition, causing a huge economic burden for individuals and society [5,6,7]. Timely intervention for strabismus promotes ocular realignment and enhances visual acuity and binocular visual function [8]. Therefore, widespread, objective, and effective screening is crucial for promptly halting the progression of strabismus [9,10]. Nevertheless, the current clinical diagnosis of strabismus is primarily based on the standardized alternating prism coverage test (PCT), which necessitates manual measurements by the clinician. The diagnostic accuracy of this method is influenced by a multitude of subjective and objective factors, including the clinician’s professional conduct, the patient’s level of cooperation, and alterations in environmental conditions.

The aforementioned issues present a risk of missing the optimal treatment window for patients with strabismus. Moreover, in developing countries, limited medical resources impede the feasibility of widespread screening using traditional methods. To address this challenge, in recent years, automatic screening based on eye tracking has begun to provide objective diagnoses for strabismus and alleviate medical burdens [11,12,13,14]. To date, several research findings have demonstrated state-of-the-art techniques capable of detecting strabismus with acceptable accuracy [15,16,17,18,19,20,21,22,23,24,25]. For example, Nixon et al. [26] presented an automated method for screening strabismus (“Strabismus screening Test using Augmented Reality and Eye-tracking”: STARE). The patient’s task was to fixate on a physical target for the 60 s with their eye movement being tracked. The results demonstrated that STARE could detect moderate and severe cases of horizontal strabismus with reasonable (87%) sensitivity, indicating its potential for effective non-invasive screening. Zheng et al. [27] developed a deep learning (DL) model for screening referable horizontal strabismus based on primary gaze photographs, using clinical assessments as a reference. The model was trained on 9402 images and demonstrated an accuracy of 95% in diagnosing referable horizontal strabismus. Chen et al. [28] proposed recognizing strabismus using eye-tracking data and convolutional neural networks. (CNNs). The eye movements of the subjects were recorded and transformed into gaze deviation (GaDe) images, which were then processed through a CNN that has been pretrained on the ImageNet dataset. This method was tested on a dataset comprising both strabismic and normal subjects, with a maximum accuracy of 95.2%.

Though current state-of-the-art works had taken significant steps to implement widespread strabismus screening, some key factors still required further research. First, commercial eye tracking systems, including stationary and portable forms [29,30], were not financially affordable for widespread strabismus detection, and some stationary systems required considerable experiment space, making widespread screening even more difficult to scale up. Second, many of the ocular features extracted by artificial intelligence (AI) algorithms, for example those extracted by machine learning (ML) and DL, were not fully interpretable physiologically, causing difficulty for biomedical staff to understand and assess the results yielded from models, even when using heatmaps [31]. Third, current examination protocols did not monitor subjects’ continuous eye motion in the wide field. Therefore, they were not capable of detecting occasional strabismus, which was of significance in identifying early-stage patients.

**Figure 1:**
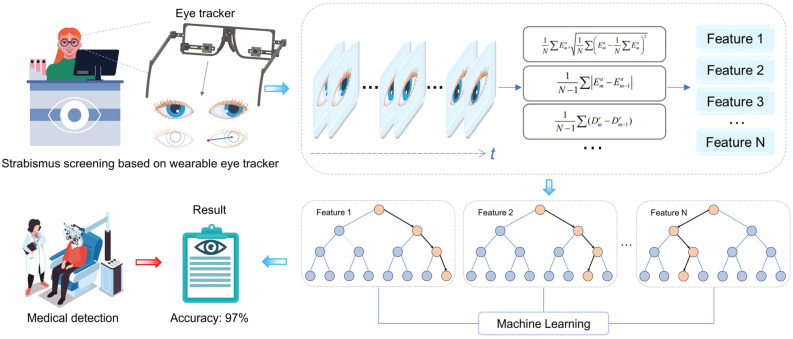
Description of the methodology.

To address the challenges explained above, this article proposes and designs a portable, wearable eye-tracking system for strabismus screening. Aiming for widespread screening application, a self-developed wearable eye tracker is assembled to capture continuous eye movement in the whole field of view using a specially designed visual guide [32]. A 16-dimensional eigenvector of the subject’s eye movement pattern is extracted and classified using the ratio of the modal length to the palpebral fissure length of the pupil-canthus vector projected onto the palpebral fissure vector, as shown in Figure 1. In comparison to previous studies, as illustrated in Table 1, our protocol has been shown to effectively induce uncoordinated eye movements in patients with intermittent strabismus by guiding continuous large-range eye movements over a wide range of visual fields, thereby demonstrating an efficient screening capability. The screening process extracts ocular movement characteristics with pronounced physiological interpretability, and we provide the weight ratio of these features in the final generated results, which are pivotal for subsequent decision-making by the physician following the initial screening [33]. Furthermore, the designed visual guidance does not require an alternate eye shading process. Therefore, the whole examination procedure does not rely on the presence of biomedical experts, lowering both time consumption and biomedical resources. The experimental results show that the system has an accuracy of 97.1% in the diagnosis of strabismus among 70 subjects, under diverse conditions (e.g., illuminations and spatial locations), supporting the high accuracy, robustness, and efficiency of the automated strabismus screening.

This paper is an extended and revised version of our previously published conference paper [22]. In the earlier work, we introduced a novel wearable eye-tracking system designed to screen for strabismus by analyzing eye movement patterns using pupil-canthus vectors. The conference paper focused on the proof of concept and initial results. The new work improves the pupil extraction algorithm, significantly reduces the feature extraction errors caused by occlusion, noise and light variations, and improves the accuracy of feature extraction. It also achieves clinical interpretability by explicitly linking the extracted features to physiological parameters through SHAP (SHapley Additive exPlanations), which comprehensively explores the clinical implications and contributes to medical knowledge beyond diagnostic applications. Furthermore, extended experimental validation under different conditions confirms the system’s adaptability, reliability and robustness in real-world environments, demonstrating its potential for use in non-clinical environments (e.g., schools or community health centers).

Section 2 presents and describes the eye-tracking device, the new methodology, and the experimental paradigm. Section 3 evaluates the classification performance and robustness of the strabismus screening system. Section 4 discusses some issues of interest. Section 5 presents the concluding remarks.

## 2. Materials and Methods

The system is devised to capture the position of the eye canthus and the pattern of movement of the pupil centers of the subjects, with a view to identifying the disease. In the first instance, an eye-tracker records an infrared, greyscale image of the subject’s eye. Subsequently, we propose an optimized pupil extraction algorithm to accurately extract the pupil data. Concurrently, the coordinates of the inner and outer canthus of the eye are introduced in this study to provide a precise basis for comparison of the subject’s strabismus. Ultimately, based on the set of eye image parameters, we acquire a feature vector set of 70 subjects containing 16 feature vectors, and employ the “leave-one-out” method to combine various ML models for classification, thereby obtaining the final classification performance.

### 2.1. Eye Tracker

**Figure 2:**
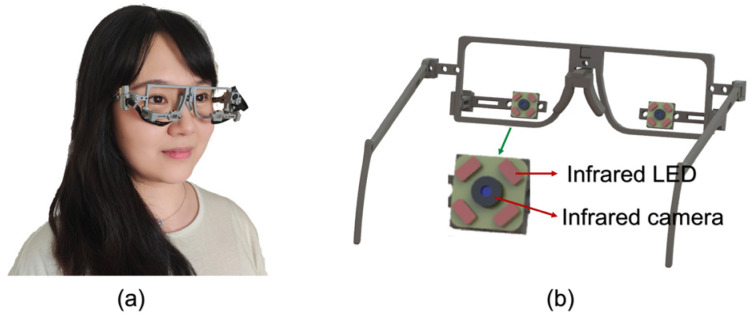
(**a**) Wearing diagram of the eye tracker. (**b**) Mechanical structure diagram of eye tracker.

In this study, we use a wearable eye tracker prototype developed by our research group and a laptop computer to perform data collection. The eye tracker prototype is composed of a 3D-printed glasses frame (printed by Creality’s CR-6 SE, Creative 3D Technology Co., Ltd., Shenzhen, China) and two small near-infrared cameras equipped with four infrared LED lights (Huibo vision Jie technology Co., Ltd., Shenzhen, China), with a total cost of 40 GBP. The camera has a frame rate of 30 Fps, 12 million pixels, a field of view of 80 degrees, and a focal length of 3.95 mm, which enables effective tracking of the subject’s eye movements. The structural and wearing diagrams of the eye-tracker model are presented in Figure 2.

### 2.2. An Optimised Pupil Extraction Algorithm

**Figure 3:**
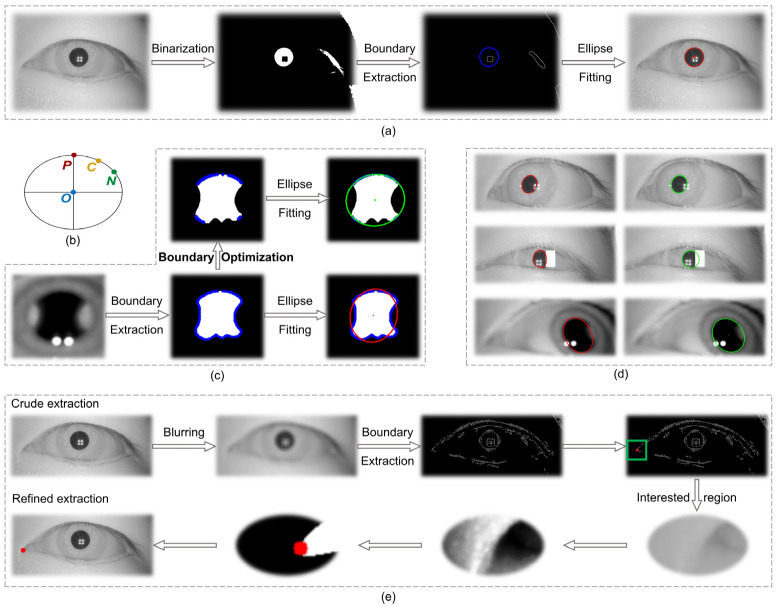
(**a**) Calculation diagram of pupil center. (**b**) Schematic diagram of ellipse boundary points. (**c**) Optimizing boundary points by three constraints to accurately extract pupil ellipse. (**d**) Comparison of the effects of traditional methods and optimization methods. (**e**) Calculation diagram of eye canthus.

The process of solving for the pupil center P in the image coordinate system included image preprocessing, binarization, boundary extraction, and ellipse fitting, as shown in Figure 3a. In eye-tracking techniques, the use of an infrared light source is essential for illumination purposes. However, this source produces primary and multilevel Pulchin spots, which, if they appear on the boundaries of the infrared and the iris, inevitably affect the extraction of the pupil boundary. The extraction of irregular pupil boundaries will, in turn, result in poor fitting of the ellipse using the conventional method. In addition to the impact of the Pulchin spot, the extraction of the pupil boundary is also influenced by the direction of gaze and the presence of eyelashes.

This study proposes a novel method for extracting the boundary points of a pupil image, utilizing three boundary point constraints. The screened boundary points are then employed in a fine extraction of the pupil ellipse. In comparison to traditional pupil ellipse extraction techniques, this method effectively addresses the distortion issues associated with pupil boundary extraction, caused by the occlusion of the pupil boundary by the Pulchin spot, eyelashes, and gaze direction. The proposed method is capable of accurately extracting the pupil center and radius.

The objective of this method is to propose three constraints for screening the extracted boundary points following the completion of pupil image preprocessing, binarization, and boundary extraction. The screened boundary points are then refitted to a least squares ellipse, and the data for the center, the long and short axes, and the inclination angle of the ellipse are summed up.

At this juncture, it is necessary to ascertain whether each boundary point meets the requisite conditions individually. For the sake of illustration, let us assume that the current point (current) is C (x_c_, y_c_), the previous point (previous) is P (x_p_, y_p_), the next point (next) is N (x_n_, y_n_), and the center of the ellipse obtained through the coarse extraction of the traditional method is O (x_o_, y_o_), as shown in Figure 3b. The restrictions are as follows:

#### 2.2.1. Direction of Curvature at Boundary Points

The pupil boundary can be approximated as an ellipse with a roundness of approximately zero. Therefore, the direction of the tangent line to the current point C should be approximately perpendicular to the line of OC. When the PN line is considered as the tangent to point C, the absolute value of the dot product of the vector $\vec{\mathrm{OC}}$ and vector $\vec{\mathrm{PN}}$ should be very small, as set forth here:

(1) $$\left|\vec{\mathrm{OC}}\cdot \vec{\mathrm{PN}}\right|=\left|\left(x_{c}-x_{o},{\;y}_{c}-y_{o})\cdot (x_{n}-x_{p},{\;y}_{n}-y_{p}\right)\right|<\;0.3$$

#### 2.2.2. Convexity of Boundary Points

It is required that the concavity and convexity of the locations of the points on the ellipse remain unaltered. Thus, it can be deduced that the difference between vector $\vec{\mathrm{CN}}$ and vector $\vec{\mathrm{PC}}$ should point to the center of the ellipse, i.e., make an obtuse angle with vector $\vec{\mathrm{OC}}$, with a cosine value of less than 0. This value is set here:

(2) $$\cos\;∠\;\left(\vec{\mathrm{CN}\;}-\vec{\mathrm{PC}},\vec{\;\mathrm{OC}}\right)=(\vec{\mathrm{CN}\;}-\vec{\mathrm{PC}})\cdot \vec{\;\mathrm{OC}}/(\vec{\mathrm{CN}}-\vec{\mathrm{PC}})\cdot \vec{\;\mathrm{OC}}\;<\;0$$

#### 2.2.3. Extreme Points in the Boundary Region

The process of least squares fitting is susceptible to influence from extreme values, both large and small. Therefore, it is necessary to remove some extreme points to ensure the integrity of the fitting process. Once the coarse extraction of the ellipse has been completed, the long and short axes (a, b) can be obtained. Subsequently, it is necessary to ensure that the length of the line segment OC is larger than the short axis length b and yet smaller than the long axis a. In order to prevent an excessive number of points from being deleted, a smaller value of ∆ (here set at 3 pixels) can be added to the long axis restriction condition:

(3) $$b\;<\;\left|\vec{\mathrm{OC}}\right|\;<\;a+∆$$

All boundary points are subjected to the three aforementioned constraints. Those that satisfy the requisite conditions are retained, whereas those that do not are discarded. Subsequently, the boundary points that satisfy the conditions are subjected to an ellipsoid fit to obtain the refined extracted ellipsoid parameters.

Conventional pupil extraction algorithms incorporate all boundary points (both pupil and non-pupil) into the elliptic least squares fitting process. However, the inclusion of non-pupil boundaries can lead to an amplification of the fitting error, as evidenced by the red ellipse in Figure 3c, whose distance from the true boundary is a clear indication of this error. The three constraints proposed in this study are designed to address the anomalous boundary points (non-pupil boundaries) that may be produced by Pulchin spots or other factors. By screening out these anomalies (as illustrated in the boundary optimization process in Figure 3c), the accuracy of the pupil ellipse fitting is optimized, as evidenced by the green ellipse in Figure 3c, which effectively captures the pupil ellipse with minimal error.

The methodology proposed in this study is employed to process a number of infrared eye images, with all resulting images demonstrating favorable outcomes. Figure 3d illustrates this, with the red ellipse representing the pupil obtained through the traditional method and the green ellipse representing the pupil obtained through the proposed methodology. It is evident that the extraction accuracy of the pupil boundaries has been enhanced.

### 2.3. Experimental Paradigm

The research protocol of this experiment has been subjected to a rigorous and comprehensive review process by the Ethics Committee of Peking University First Hospital. This ensures that the research activities comply with the highest international standards of medical ethics and relevant Chinese laws and regulations. Furthermore, the rights, privacy, and security of the research participants are fully respected and protected. The study comprises 70 volunteers, 35 normal subjects, and 35 patients (one of whom has recently undergone surgery and is therefore classified as a normal individual) with exotropia, with ages ranging from 8 to 50 years. The eyes of the volunteers had all types of health statuses according to professional clinical ophthalmologists.

**Figure 4:**
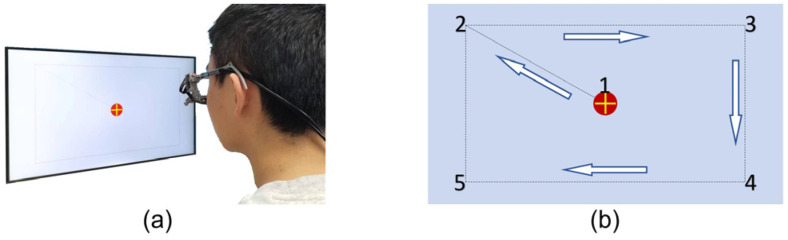
(**a**) Subject testing procedure. (**b**) Screen target point movement.

Given the extensive age range of the subjects, a variety of potential complexities may arise during the actual experiment, which include instances of children exhibiting involuntary movements during the test, fluctuations in experimental lighting, and uncertainty regarding the experimental position due to height. To emulate the circumstances typically encountered in routine strabismus screening, we do not impose certain specific constraints on the experimental parameters. The subjects are seated in a comfortable position and observe the oscillating yellow plus center light spot on the screen, as illustrated in Figure 4a. The light spot initially flashes in the center of the screen for a period of three seconds, after which it moves uniformly along a dotted line in accordance with the sequence of the number “1-2-3-4-5-2”, as shown in Figure 4b, for a total duration of 20 s. The eye tracker records the infrared grey image of the eye in real time, with 540 grey images being recorded for each eye during the experiment.

### 2.4. Feature Processing of Eye Images

#### 2.4.1. Eye Image Parameter Extraction

Strabismus cannot be accurately compared between subjects by pupil movement alone because of the lack of adequate reference points. Taking the example of two patients with disparate pupil distances, it is a common pitfall to misdiagnose an individual with a larger pupil distance as an exotropic patient based on the absolute position of the pupil center as it moves. This study introduces and normalizes the coordinates of the inner and outer canthus of the eye to allow for a more accurate assessment of strabismus by taking into account variations in individual facial structures and ensuring that eye movement data is not affected by pupil size, head position or other factors that can lead to variations in measurements [38,39]. In addition, the normalization of pupil-canthus vectors facilitates the comparison of eye movement patterns between subjects, which is critical for large-scale screening projects where consistency in data interpretation is essential.

In the image coordinate system, the precise extraction processes of the canthus (taking the inner canthus of the eye for example) are blurring, boundary extraction, coarse extraction of corner points, histogram equalization of the region of interest, binarization, and canthus point extraction as shown in Figure 3e. Finally, the fine extraction of the coordinates of the inner canthus of the eye, $C_{I}$ (x_Ci_, y_Ci_), and the outer canthus, $C_{O}$ (x_Co_, y_Co_), is achieved.

**Figure 5:**
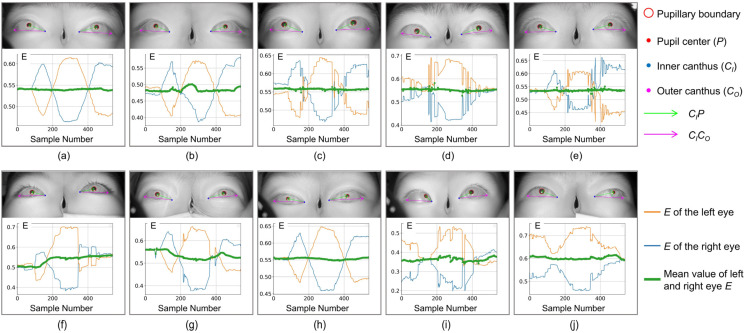
Binocular image and the graph of $E_{m}^{l}$ (blue curve), $E_{m}^{r}$ (orange curve), $E_{m}^{a}$ (green curve), where (**a**–**e**) are normal individuals and (**f**–**j**) are strabismus patients.

The ratio of the projection length of vector $\vec{C_{I}P}$ in the direction of vector $\vec{C_{I}C_{O}}$ to the mode length $\vec{C_{I}C_{O}}$ is used as the dimensionless parameter value E for each eye image sampling, as described in Equation (4). For each subject, the left eye parameter group $E_{m}^{l}$, the right eye parameter group $E_{m}^{r}$, and their mean values $E_{m}^{a}$ are obtained. $E_{m}^{l}$, $E_{m}^{r}$, and $E_{m}^{a}$ correspond to the blue, orange, and green curves in Figure 5, respectively (the red is the curve after filtering and smoothing the median value of $E_{m}^{a}$). If there is no clear indication in the following, m = 1, 2,…, N, N is 540.

(4) $$E=\vec{C_{I}P}\cdot \vec{C_{I}C_{O}}/{\left|\vec{C_{I}C_{O}}\right|}^{2}$$

#### 2.4.2. Feature Vector and Classification

Based on the set of eye image parameters of a subject, the method of calculating the eigenvector {$F_{i}^{k}$, i = 1, 2,…, 16} and its relevance can be analyzed in depth. k denotes the subject number and i denotes the eigenvalue number.

Ideally, a strabismus patient will have an $E_{m}^{a}$ that deviates from a normal person’s $E_{m}^{a}$ range due to an uncontrolled deviation of one eye to the inner or outer canthus of the eye. Additionally, when a normal person observes an object whose trajectory is parallel to the plane of the face, the relative position of the pupils of both eyes remains basically unchanged. Therefore, the mean value, standard deviation, maximum value, and minimum value of $E_{m}^{a}$, and the mean and standard deviation of the absolute value of the primary difference of $E_{m}^{a}$ are taken as part of the eigenvalues, as shown in Equations (5)–(8).

(5) $$\left\{F_{1},F_{2}\right\}=\left\{\frac{1}{N}\sum E_{m}^{a},\;\sqrt{\frac{1}{N}\sum {\left(E_{m}^{a}-\frac{1}{N}\sum E_{m}^{a}\right)}^{2}}\right\}$$

(6) $$\left\{F_{3},F_{4}\right\}=\left\{\max\left(E_{m}^{a}\right),\;\min(E_{m}^{a})\right\}$$

(7) $$F_{5}=\frac{1}{N-1}\sum \left|E_{m}^{a}-E_{m-1}^{a}\right|$$

(8) $$F_{6}=\sqrt{\frac{1}{N-1}\sum {\left(\left|E_{m}^{a}-E_{m-1}^{a}\right|-F_{5}\right)}^{2}}\;m=2,\;\ldots ,\;N$$

The subjects’ face shape, pupil distance, sitting angle, screen distance, and other factors greatly interfere with the classification results, so the inner canthus distance and outer canthus distance are introduced into the feature vector, where $C_{I}^{l}{,\;C}_{O}^{l}$ represent the inner and outer canthus coordinates of the left eye and $C_{I}^{r}{,\;C}_{O}^{r}$ represent the right eye in the image coordinate system, as shown in Equation (9).

(9) $$\left\{F_{7},F_{8}\right\}=\left\{\left|\vec{C_{I}^{l}C_{I}^{r}}\right|,\left|\vec{C_{O}^{l}C_{O}^{r}}\right|\right\}$$

**Figure 6:**
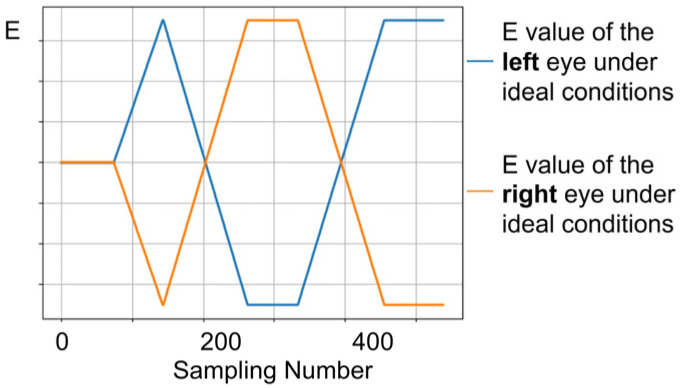
Diagram of the standard values of the set of parameters for normal movement of both eyes under ideal conditions. This chart only shows trends, so the vertical axis is meaningless.

In comparison to individuals with normal vision, patients with strabismus may exhibit varying degrees of asymmetry in ocular movement during eye-tracking tasks. Therefore, the comparison of binocular parameters is equally crucial. In the ideal state, the standard values of the normal binocular movement parameter groups are $E_{m}^{l,s}$ (blue curve) and $E_{m}^{r,s}$ (orange curve), respectively, as shown in Figure 6.

First, the mode of the first 70 parameters with the subject staring at the center of the screen is calculated, and this mode is introduced into the feature vector as the initial value of the irregular movement of the eyes, as shown in Equation (10).

(10) $$\left\{F_{9},F_{10}\right\}=\left\{\mathrm{mode}\left(E_{m}^{l}\right),\mathrm{mode}\left(E_{m}^{r}\right)\right\},m=1,\;2,\;\ldots ,\;70$$

Subsequently, the difference $D_{m}^{l},\;D_{m}^{r}$ between the subject’s monocular movement and the standard value are used as the characterization of the irregular movements of the left and right eyes, as shown in Equation (11).

(11) $$\left\{D_{m}^{l},D_{m}^{r}\right\}=\left\{E_{m}^{l}-E_{m}^{l,s}\;,\;E_{m}^{r}-E_{m}^{r,s}\right\}$$

The standard deviation of the absolute value of $D_{m}^{l},{\;D}_{m}^{r}$, and the mean and standard deviation of the first difference of $D_{m}^{l},{\;D}_{m}^{r}$ are introduced as eigenvalues into the feature vector, as shown in Equations (12)–(16).

(12) $$\left\{F_{11},F_{12}\right\}=\left\{\frac{1}{N}\sum \left|D_{m}^{l}\right|\;,\;\frac{1}{N}\sum \left|D_{m}^{r}\right|\right\}$$

(13) $$F_{13}=\frac{1}{N-1}\sum (D_{m}^{l},D_{m-1}^{l})\;m=2,\ldots ,N$$

(14) $$F_{14}=\frac{1}{N-1}\sum (D_{m}^{r},D_{m-1}^{r})\;m=2,\ldots ,N$$

(15) $$F_{15}=\sqrt{\frac{1}{N-1}\sum {\left[\left(D_{m}^{l}-D_{m-1}^{l}\right)-F_{13}\right]}^{2}}$$

(16) $$F_{16}=\sqrt{\frac{1}{N-1}\sum {\left[\left(D_{m}^{r}-D_{m-1}^{r}\right)-F_{14}\right]}^{2}}$$

After the computation of the eigenvector {$F_{i}^{k}$, i = 1, 2, …, 16}, the “leave-one-out” method is used to combine various ML models for classification, such as RF, Logistic Regression (LR), Decision Trees (DT), SVM, K-Nearest Neighbors (kNN), Naive Bayes (NB), Gradient Boosting Decision Trees (GBDT), and Adaptive Boosting (AB).

We have a total of 70 feature vectors (70 subjects). One feature vector is used each time for the classification test and the remaining 69 are used for training. Therefore, we can get 70 classification results and compare them with the labeled values to obtain the final classification performance.

## 3. Results

**Figure 7:**
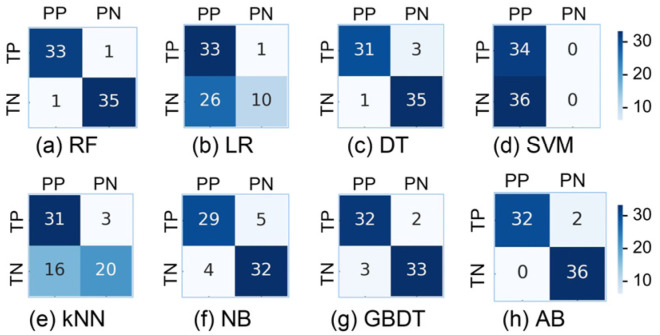
Classification confusion matrix diagram of multiple classification models. Two rows of the matrix are the number of patients and normal individuals, and two columns are the predicted number of patients and normal individuals. In this case, TP represents the true patient, TN represents the true normal people, PP represents the predicted patient, and PN represents the predicted normal people.

In this study, we use a variety of models to classify feature vectors, and the results are shown in Figure 7 and Table 2. The RF model shows better results, with strong classification of strabismus patients (P = 0.97, R = 0.86, F1 = 0.92) and normal subjects (P = 0.85, R = 0.97, F1 = 0.91). The results show that the accuracy rate of screening for strabismus patients is as high as 97.1%, and the model has strong generalization screening ability. In addition, the recall rate of normal people also reaches a high level of 97.1%, which indicates that the misdiagnosis rate is very low.

The occurrence of strabismus is not a continuous process, thus making it difficult to distinguish some strabismus patients from normal individuals in terms of external characteristics of the eyeball in a resting state. Similarly, in the early stages of the disease, there is no obvious strabismus feature for screening.

Among the 10 subjects shown in Figure 5, if observed only from static binocular images, except for subjects (i) and (j) where obvious features of esotropia or exotropia can be observed, there is almost no significant difference in subjects (a–h), indicating that the screening method based on static gaze images is not sufficient to achieve good screening results for patients with weak strabismus and intermittent strabismus.

For patients (f) and (g) in Figure 5, it can be seen from $E^{a}$ (green line) and the infrared image in the figure that at the beginning of the experiment, both eyes show good consistency without obvious strabismus. However, during the visual induction process, due to diseases, eye movements are not synchronized, $E^{a}$ changes significantly, and strabismus features are prominent. During full-field eye movement, the inconsistency of binocular coordination ability can be effectively highlighted.

It is worth noting that the consistency of binocular coordinated movement characterized by the green line alone is not sufficient to achieve the screening of patients with strabismus. The patient’s $E^{a}$ in Figure 5h has maintained a relatively stable state, while $E^{a}$ of normal individuals in Figure 5e also shows relatively significant changes during visual induction (possibly caused by blinking or distraction). Our method effectively solves the above problems by introducing many features derived from the pupil angle vector (such as differentiation, mean, variance, etc.), greatly improving the screening accuracy of the model.

Comparing the healthy subjects (c), (d), and (e) in Figure 5, despite using the same screening method, their test performance differs significantly. Although the binocular coordination curves all show three peaks and valleys, the positions and duration of the peaks and valleys are different, making it difficult to accurately correspond with specific stages in the testing process. In medicine, a possible explanation is that due to the non-coincidence of the optical and visual axes when observing objects, different individuals in different age groups exhibit different Kappa angles (the angle between the optical and visual axes) when observing objects at the same angle. This individual difference poses a severe challenge to the generalization performance of screening algorithms. In this regard, the RF algorithm can effectively overcome the limitation of single-classifier overfitting and weaken the impact of individual differences on the model’s generalization ability in strabismus screening tasks. In addition, this method exhibits good noise suppression ability and a certain robustness in the face of some isolated noise points in the binocular coordination curve.

**Figure 8:**
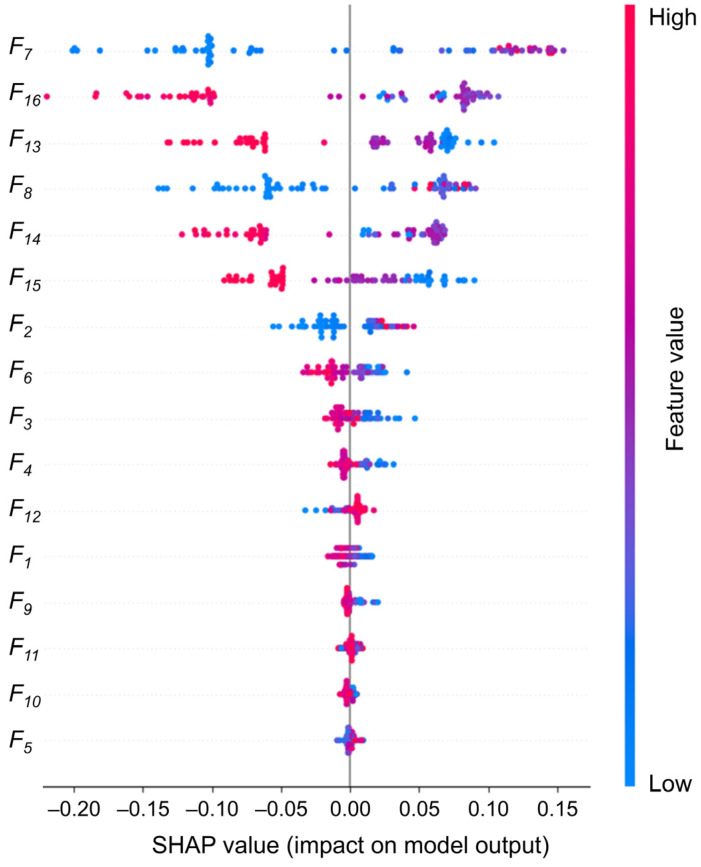
Density scatterplot on 16 feature weights.

**Figure 9:**
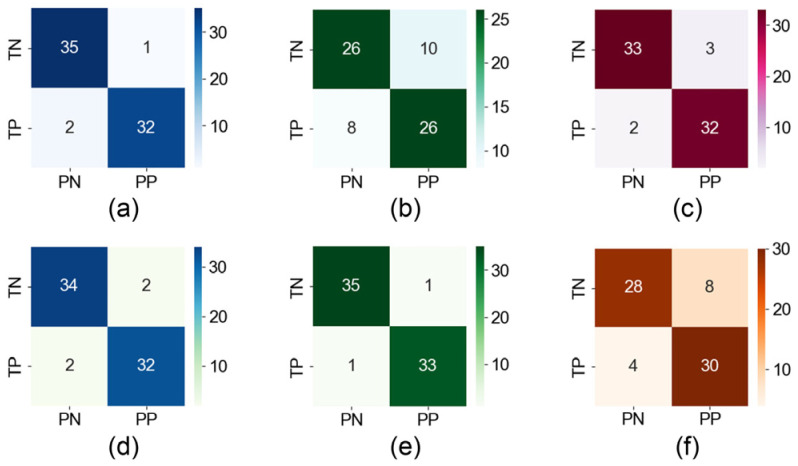
Classification matrix with reduced eigenvalues. (**a**) 95.7% accuracy for deleting 10 features with low-weight values. (**b**) 74.3% accuracy for deleting 6 features with high weight values. (**c**) 92.9% accuracy for deleting $F_{7}$ and $F_{8}$. (**d**) 94.3% accuracy for deleting $F_{13}\;$ and $F_{14}$. (**e**) 97.1% accuracy for deleting $F_{15}$ and $F_{16}$. (**f**) 82.9% accuracy for deleting $F_{13}$, $F_{14}$, $F_{15}$, and $F_{16}$.

In addition to the performance metrics, we conduct an in-depth analysis of the 16 eigenvalue contributions using SHAP values, as illustrated in Figure 8. This analysis aims to gain a more comprehensive understanding of the decision-making process of the RF model and to provide an effective reference for the physician’s further diagnosis. The weights of the eigenvalues are calculated, and it is found that five eigenvalues have weight values above 0.1. F15 is slightly lower (0.088), and the remaining eigenvalues have weight values below 0.05. Following the deletion of the 10 features with low weights and their reclassification, the accuracy rate is found to be 95.7%, which still represents a high level of classification performance. However, after deleting the six most important features, the accuracy rate decreases significantly to 74.3%, as shown in Figure 9a,b. Consequently, our focus shifts to interpreting the six eigenvalues that had the greatest impact.

The distance between the inner and outer corners of the eye is intimately associated with eye position and symmetry, representing a fundamental structural feature of the eye. It can be seen that the medial canthus distance (0.229) and the lateral canthus distance (0.135) have a greater concentration of high SHAP values, indicating that these two features consistently have a positive effect on the model output when predicting strabismus. This highlights their clinical relevance in detecting abnormal eye positions. For example, internal strabismus may result in a reduction in the distance between the eyes. In the meantime, the first-order difference features of eye movements ($F_{13}$: 0.118, $F_{14}$: 0.115, $F_{15}$: 0.088, $F_{16}$: 0.160) represent the dynamic features, which are widely distributed in the SHAP plots. This indicates that the transient changes in eye movements play an important role in model prediction and that irregular fluctuations drive the prediction of strabismus. Notably, the weight assigned to $F_{16}$ is markedly higher than that assigned to $F_{15}$, which may indicate that the instability of the right eye during dynamic tasks has a more pronounced impact on the prediction of strabismus.

In Figure 9, we conduct a pair-wise removal of the aforementioned six key features before reclassification. The results demonstrate that the removal of $F_{7}$ and $F_{8}$ results in the most significant decline in accuracy, reaching 92.9%, suggesting that the structural characteristics of the eye play a more pivotal role in the model’s classification.

Furthermore, additional eye movement-related features contribute relatively little but provide supplementary diagnostic insight, enabling the model to capture more subtle patterns of ocular deviation, which aids in classification. This robust combination of static and dynamic features ensures that the RF model not only achieves high accuracy but also maintains strong clinical relevance, providing reliable physiological references for the early detection and screening of strabismus in a variety of patient populations.

**Figure 10:**
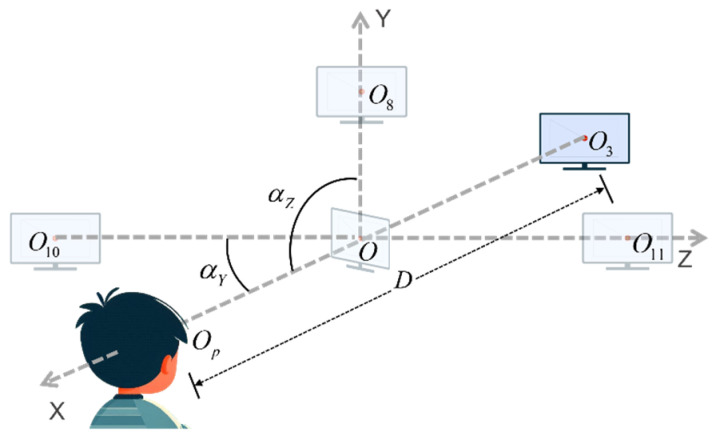
Schematic diagram of robustness test experiment.

Finally, we define the experimental scope of this set of paradigms based on real experimental conditions with a sample size of 70 subjects. During the actual screening process, fluctuations in the variables are observed, primarily in the subject, with the monitor remaining stationary. Here, to more precisely explain the range of parameters for the experimental setting, we assume that the subject would remain stationary while the position of the monitor changes, i.e., the position of the center of the monitor changes relative to the center of the subject’s pupil. The initial position of the center of the monitor is designated as O, and the center of the subject’s pupils is designated as $O_{p}$. At this point, the subject is positioned at eye level. The center of the monitor is set to be $O_{i}$ in the experiment, as shown in Figure 10. The delineated range of each parameter is presented in Table 3, with the initial situation indicated in the first row of data. The experimental environment permits a range of light conditions, from complete darkness to full daylight. Additionally, minor random fluctuations in the subjects’ body and head movements are acceptable.

## 4. Discussion

In this study, the advantages of a wearable eye-tracking camera for the detection of intermittent strabismus are demonstrated, addressing some of the limitations inherent in traditional screening methods.

Wearable eye-tracking cameras are employed to capture images of the eyes. While high-definition cameras are effective at capturing facial details such as eye corners and pupils, they require controlled settings to ensure accurate and consistent data collection. In contrast, goggle-style cameras offer a more practical and versatile solution for capturing eye movement data during natural activities. Their portability and ease of use make wearable devices more suitable for widespread screening, especially in areas with limited medical facilities. Furthermore, the device’s affordable price (approximately 40 GBP) and simple operational design, coupled with a user-friendly experimental paradigm (no ocular occlusion required), enhance its potential for large-scale implementation.

In addition to the benefits derived from the hardware, our methodology involves the extraction of a rich set of ocular features for the purpose of analyzing the data pertaining to eye movement. Although sophisticated algorithms such as ML are capable of extracting higher-dimensional ocular features, the lack of a clear physiological interpretation of these features presents a challenge for physicians who rely on such insights to diagnose and treat strabismus. The discrepancy between algorithm output and clinical application hinders the integration of ML/DL-based methods into everyday practice. In this study, the correlation of 16 feature vectors with known physiological parameters enables physicians to make subsequent decisions and facilitates the identification of new diagnostic markers, thereby contributing to a more profound comprehension of the pathophysiology of strabismus. In addition, conventional screening techniques frequently prove inadequate for the detection of the erratic, episodic eye movements that typify intermittent strabismus. By utilizing a wide-angle continuous moving cross-sign paradigm, however, it is possible to elicit and capture these events with greater efficacy, thereby facilitating a more accurate diagnosis than would be possible with traditional methods.

Finally, the inclusion of data from 70 volunteers with varying age groups and types of strabismus reinforces the dependability of the findings. The benchmark for comparison is the clinical diagnosis by an experienced ophthalmologist using the standard prismatic alternating coverage test (PCT), which is widely regarded as a reliable method for strabismus evaluation [40]. By employing this dependable reference standard, the precision of our system is substantiated by clinically confirmed cases, thereby guaranteeing the reliability of the outcomes.

Our study uses a spectacle-like wearable eye tracker for high-precision strabismus screening. However, future wearable technology may further improve portability and user compliance. Smart contact lenses that embed sensors on the ocular surface offer a promising alternative for continuous, unobtrusive eye movement monitoring in natural environments. They could detect subtle or intermittent strabismus patterns that controlled settings might miss. Recent advances in flexible electronics and miniaturized sensors [41,42] support the feasibility of integrating gaze tracking functions into contact lenses, with potential benefits in comfort and aesthetics, especially for paediatric use. However, several challenges need to be overcome before clinical implementation. Key issues include ensuring biocompatibility, achieving sufficient spatial resolution for accurate pupil tracking, and managing power consumption for prolonged use. In addition, data processing algorithms need to be refined to account for the unique noise and motion artefacts of lens-based systems. Our current methodology—which emphasizes robust feature extraction and normalization (e.g., pupil-canthus vectors)—could serve as a basis for interpreting data from smart contact lenses. Future research could explore hybrid systems that combine wearable glasses for calibration with contact lenses for long-term monitoring, thereby balancing accuracy and practicality.

In the future, medical artificial intelligence (AI) has the potential to enhance the accuracy and efficiency of strabismus screening. By analyzing large volumes of ocular movement data, AI can automatically detect subtle changes in strabismus, enabling early detection of symptoms that may be overlooked by traditional methods, facilitating earlier intervention and treatment. AI algorithms can also assist clinicians in accurately assessing the stage of strabismus based on eye movement patterns and physiological changes, helping to develop personalized treatment plans. Furthermore, AI, through the application of deep learning models to extensive clinical data, has the potential to uncover potential biomarkers that may be undetectable with traditional methods, thus improving early diagnostic capabilities. Additionally, AI can reveal correlations between strabismus and other diseases, such as neurological disorders (e.g., Parkinson’s, Alzheimer’s) and metabolic conditions (e.g., diabetes), offering new insights for early screening and intervention. Furthermore, it can assist in identifying genetic, environmental, or lifestyle factors that influence strabismus development, thus advancing the field of etiological research. These findings not only enhance the understanding of strabismus pathophysiology, but may also stimulate interdisciplinary research and contribute to the progress of medical diagnostics and treatment. As AI technology continues to evolve, its application in ophthalmology, particularly in strabismus screening and treatment, is expected to become more widespread and mature [43,44].

## 5. Conclusions

In this research, we present an innovative strabismus screening system based on eye-tracking technology, achieving a high screening rate of up to 97.1%. The protocol is more likely to induce intermittent strabismus and demonstrates a set of eye movement characteristics with strong physiological interpretability, which informs physicians’ diagnoses. Compared with other automated strabismus diagnostic systems, our system is characterized by high accuracy and high robustness, and it is expected to achieve widespread popularity.

## Figures and Tables

**Table 1:** Related works.

Studies	Devices	Number of Experimenters	Experimental Paradigm	Method	Accuracy
Saisara, et al. [16]	Desktop eye tracker	46 N and 4 P	Four-point gaze method (no occlusion process)	Pupillary distance is extracted.	N/A
Miao, et al. [18]	VR based on infrared camera	17 P	Monocular alternate occlusion	Eyeball diameter is extracted.	95%
Nixon, et al. [26]	Augmented Reality (AR) based on wearable eye-tracker	7 N and 19 P	Monocular alternate occlusion	The gaze position is extracted.	87%
Zheng, et al. [27]	N/A	6070 images	Collection of orthoptic photographs of children	Feature extraction through deep convolutional neural networks (DCNNs).	95%
Chen, et al. [28]	Stationary eye-trackers	25 N and 17 P	Nine-point gaze method (no occlusion process)	Feature extraction through convolutional neural network (CNN).	95.2%
Valente, et al. [34]	Camera (50 cm away)	7 P	Monocular alternate occlusion	Pupil center and limbus coordinates are extracted.	87%
Khumdat, et al. [35]	Camera (100 cm away)	103 subjects	N/A	The central corneal light reflex ratio is extracted.	94.17%
Yang, et al. [36]	Infrared camera	90 P	Observation of the cornea and pupil of the occluded eye	The location of the corneal limbus, pupil, and corneal light reflection are extracted.	90%
Dallyson, et al. [37]	Camera (50 cm away)	30 N and 15 P	Single point gaze	Positioning the eyes using support vector machine (SVM).	94%
Our work	Wearable eye tracker	35 N and 35 P	Continuous binocular movement within an extensive field of vision (no occlusion process)	16 feature vectors related to physiological parameters of the eye.	97.1%

**Table 2:** Classification accuracy of each model (%).

Mode	RF	LR	DT	SVM	kNN	NB	GBDT	AB
Patient precision	97.1	97.1	91.2	100	91.2	85.3	94.1	94.1
Overall accuracy	97.1	61.4	94.3	48.6	72.9	87.1	92.9	97.1

**Table 3:** Parameter setting for experimental conditions.

Experimental Variables	D (cm)	$\alpha_{Z}$ (°)	$\alpha_{Y}$ (°)	$\vec{OO_{i}}$ (cm)
N/A	75	90	90	(0, 0, 0)
D & $\vec{OO_{i}}$(X)	30–90	90	90	(45, 0, 0)–(−15, 0, 0)
$\alpha_{Z}$	75	60–120	90	(0, 0, 0)
$\alpha_{Y}$	75	90	60–120	(0, 0, 0)
$\vec{OO_{i}}$(Y)	75	90	90	(0, 30, 0)–(0, −30, 0)
$\vec{OO_{i}}$(Z)	75	90	90	(0, 0, 30)–(0, 0, −30)

D—The distance from O_i_ to the plane where the subject’s pupils are located, $\alpha_{Z}$—Angles lying in the xOy plane, $\alpha_{Y}$—Angles lying in the xOz plane, and all angles are rotated in a clockwise direction, with the O_i_O_p_ designated as the initial edge, $\vec{OO_{i}}$ (X)—The x-axis coordinates of vector $\vec{OO_{i}}$, $\vec{OO_{i}}$ (Y) and $\vec{OO_{i}}$ (Z) are the same as before.

## Data Availability

The eye image data of the subjects used in this study cannot be shared due to privacy.
